# Increased Sensitivity to Ionizing Radiation in a Relevant Subset of Patients with Cancer and Systemic Lupus Erythematosus

**DOI:** 10.3390/cells14080569

**Published:** 2025-04-09

**Authors:** Hannah Schenker, Lukas Kuhlmann, Dorothee Kaudewitz, Barbara Schuster, Sabine Semrau, Charlotte Schmitter, Raphaela Voigt, Ricarda Merten, Hans Geinitz, Rainer Fietkau, Sebastian Böltz, Georg Schett, Luitpold V. Distel

**Affiliations:** 1 Department of Internal Medicine 3—Rheumatology and Clinical Immunology, Universitätsklinikum Erlangen, Friedrich-Alexander-Universität Erlangen-Nürnberg, 91054 Erlangen, Germany; 2 Department of Internal Medicine 1—Gastroenterology, Pneumology and Endocrinology, Universitätsklinikum Erlangen, Friedrich-Alexander-Universität Erlangen-Nürnberg, 91054 Erlangen, Germany; 3 Deutsches Zentrum Immuntherapie, Universitätsklinikum Erlangen, Friedrich-Alexander-Universität Erlangen-Nürnberg, 91054 Erlangen, Germany; 4 Department of Radiation Oncology, Universitätsklinikum Erlangen, Friedrich-Alexander-Universität Erlangen-Nürnberg, 91054 Erlangen, Germany; 5 Comprehensive Cancer Center Erlangen-Europäische Metropolregion Nürnberg, 91054 Erlangen, Germany; 6 Internal Medicine V, Haematology, Oncology and Rheumatology, Heidelberg University Hospital, 69120 Heidelberg, Germany; 7 Department of Radiation Oncology, Ordensklinikum Linz Barmherzige Schwestern, 4010 Linz, Austria; 8 Medical Faculty, Johannes Kepler University, 4020 Linz, Austria

**Keywords:** systemic lupus erythematosus, individual radiosensitivity, cancer, radiochemotherapy, side effects, dose adaption

## Abstract

It has long been hypothesized that systemic lupus erythematosus (SLE) increases radiosensitivity, but recent studies have yielded mixed results. We studied individual radiosensitivity in 70 individuals with SLE using chromosomal aberrations as biomarkers of radiosensitivity. In total, 33 patients with SLE and 37 patients with SLE and additional oncologic diseases were compared with healthy individuals and with patients with rectal and breast cancer. Individual radiosensitivity was assessed by ex vivo irradiation of G0 blood lymphocytes followed by three-color fluorescence in situ hybridization of chromosomes 1, 2, and 4. SLE patients have slightly higher background rates of chromosomal aberrations than healthy individuals and lower rates than cancer patients. Non-oncologic SLE patients show a rate of chromosomal aberrations similar to that seen in healthy individuals. The outliers in this group, who clearly show increased radiosensitivity, fall between healthy individuals and cancer patients. Patients with SLE and cancer have significantly higher chromosome aberration rates compared to healthy individuals (*p* < 0.001) and patients with isolated cancer (*p* = 0.007) or isolated SLE (*p* = 0.004). The proportion of radiosensitive patients in the oncologic SLE cohort is high, with 45% of patients showing increased radiosensitivity. There is a weak association between anti-Ro-52 autoantibodies and radiosensitivity. Based on the radiosensitivity measurement, radiation dose reduction was recommended in 11 oncological SLE patients and was successfully achieved in 5 patients by up to 21% of the dose per fraction. In the oncologic SLE cohort, a substantial portion of individuals show increased radiosensitivity.

## 1. Introduction

Systemic lupus erythematosus (SLE) is a complex autoimmune disease with a wide spectrum of cutaneous and systemic manifestations [1]. The 5-year survival rate for systemic lupus erythematosus has increased from about 40% in the 1950s to 90% in the 1980s and over 90–95% today [2]. However, as the risk of developing cancer increases with age, a growing number of SLE patients will develop malignancies. Additionally, several studies have highlighted an increased risk for cancer in patients with SLE compared to the general population [3]. Radiotherapy is still part of the first-line therapy of many tumor types; however, in SLE patients, radiotherapy is associated with a number of problems, i.e., exacerbations of the SLE, skin, and mucosa inflammation, skin and soft tissue fibrosis, wound complications, and consecutive infections. A number of case reports describe considerable worsening of the disease during radiotherapy, which consequently had to be terminated [4,5,6,7,8,9,10].

There has long been a suspicion that SLE leads to increased sensitivity to radiation [11,12,13]. A 2009 review summarized the studies on SLE and radiosensitivity, highlighting a tendency for patients with SLE to have a higher risk of increased radiosensitivity [14]. SLE patients have an increased sensitivity to UV radiation due to increased apoptosis of keratinocytes [15]. In recent years, there have been several studies on impaired DNA damage processing in SLE patients [16]. Radiotherapy (RT) also impairs proliferation and fibrosis, especially in the immediately exposed skin and the mucosa causing a reduction in capillary and small vessels [17]. This might explain why the tissue damage caused by RT and the pre-existing damage caused by SLE could lead to an increase in the development of side effects [18]. However, results of previous studies are heterogeneous and a recent review of the literature does not confirm an increased overall risk for radiation-induced toxicity [15,19,20]. Nevertheless, radiation therapy in several cases is still withheld from many SLE patients.

To improve the safety of radiotherapy in SLE patients and to avoid inappropriate exclusion of these patients from effective anti-cancer therapy, more detailed insights into the radiation sensitivity of SLE patients are needed. Determining an individual’s sensitivity to radiation is difficult because it involves not only the various DNA repair pathways, with approximately 450 genes, but also signal transduction, cell cycle control, and cell death control [21]. Chromosome analysis after ex vivo irradiation is a very laborious but reliable test [22,23,24]. This test exploits blood lymphocytes, which are always in the G0 phase of the cell cycle. These lymphocytes are then ex vivo irradiated and stimulated to undergo cell division. At this point, the lymphocytes are able to repair the X-ray-induced DNA damage, pass on the information about the damage, and go through the entire cell cycle. This is where cell fate is decided. As the cells enter metaphase, the mutations that are present are imprinted as chromosomal aberrations [23,25]. We have studied a cohort of SLE patients with and without cancer to determine their radiosensitivity by chromosomal aberration analysis after ex vivo irradiation. In patients with increased chromosomal aberrations, we investigated risk factors for increased radiosensitivity. We further studied whether dose adjustment after radiosensitivity testing is an effective way to reduce the risk of overtreatment in patients with increased radiosensitivity.

## 2. Materials and Methods

### 2.1. Retrospective Cohort of SLE Patients Investigating Undertreatment with Radiotherapy

The hospital database was searched for SLE patients and 1086 patients were identified who were treated for SLE between 2000 and 2020. These patients were searched for in the cancer registry and 64 patients with cancer (ICD10, C00-C97 without C44) were identified. The records of these patients were searched for their tumor status and their treatment. It was also determined whether radiotherapy would have been necessary and whether it had been given.

### 2.2. Prospective Patient Cohorts of SLE Patients for Individual Radiosensitivity

In total, the blood of 658 individuals was analyzed to estimate individual radiosensitivity using three-color fluorescence in situ hybridization. Overall, 37 individuals with systemic lupus erythematosus (SLE-non-onco) and 33 patients with cancer and SLE (SLE-onco) who had opted for radiotherapy treatment were compared with 215 healthy subjects, 226 patients with rectal cancer, and 147 patients with breast cancer [26]. Healthy individuals were defined to have no history of a malignant disease and to have a Karnofsky performance status of at least 90. Both cohorts of SLE patients were studied prospectively. The diagnosis of SLE was consistent with the recently published classification criteria by EULAR and ACR. The healthy individuals were 57.8% female and had a mean age of 50.8 years; the respective numbers were 29.2%/62.8 years in the rectal cancer cohort, 100%/57.3 years in the breast cancer cohort, 75.7%/49.6 years in SLE-non-onco patients, and 81.8%/56.6 years in SLE-onco patients. The mean age at SLE diagnosis was 36.7 years in the SLE-non-onco cohort and 38.0 years in the SLE-onco cohort. In the SLE-onco cohort, the time between SLE diagnosis and cancer onset was 13.8 years (range 2–47 years) (Table 1).

The most commonly used drugs in the SLE-non-onco cohort were hydroxychloroquine (45.9%), chloroquine (16.2%), and belimumab (16.2%) (Table 2). Patients with a prednisolone dose of more than 20 mg/d had to be excluded, as lymphocytes cannot be stimulated to divide to a sufficient extent under this therapy. The patients had various clinical manifestations, which included mostly joint involvement, rash, and hematological aberrations. The distribution of the detected antibodies is shown in the results. The rectal cancer group was consecutively sampled in the University Hospital of Erlangen between 2009 and 2016. Between 2006 and 2018, healthy individuals [24] and the breast cancer cohort were sampled. All blood samples were taken prior to the beginning of the radiotherapy. Parts of the rectal cancer data and healthy individuals data have been published previously [26,27]. Patient blood and SLE-non-onco patient data were collected prospectively. Therapy data for the SLE-onco group were derived retrospectively. Antibodies in the SLE-non-onco group were analyzed on a routine basis.

All patients and healthy individuals gave written informed consent to the scientific processing of their material and data. The Ethics Review Committee of the University Hospital Erlangen approved the study including the use of individual patient data. Ethics committee approval 21_19 B.

### 2.3. Three Color Fluorescence In Situ Hybridization of Chromosomes 1, 2, and 4

Overall, 9 mL blood was drawn from the patients and half was irradiated with 2 Gy of ionizing radiation (6-MV linear accelerator, Varian, Palo Alto, CA, USA) to induce chromosomal aberrations [28]. The other half was used to analyze background chromosomal aberrations. In a blood culture, lymphocytes were irradiated in the G0 phase and then stimulated with phytohemagglutinin (PHA-L pure, Biochrom, Berlin, Germany). After 44 h, colcemid was added for another 3 h to arrest the cells in metaphase. Chromosome preparation was performed according to a standard protocol. Chromosomes #1, #2, and #4 were stained in red, green, and yellow by fluorophores FITC and Rhodamine and DNA was counterstained with DAPI.

### 2.4. Image Acquisition and Analysis

Metaphases were searched automatically in the hybridized area (Metafer 4 V3.10.1, Altlussheim, Germany) by a fluorescence microscope (Zeiss, Axioplan 2, Göttingen, Germany) at 100× magnification and single metaphase images were acquired at 630× magnification. The breakage rate in each metaphase was scored with semiautomatic image analysis software (V6.1) (Biomas, Erlangen, Germany). Chromosomal aberrations were analyzed according to the underlying DNA double-strand breaks [29] by counting fragments as one break, translocations, dicentrics, and rings as two breaks and complex aberrations as the number of breaks theoretically necessary to explain the resulting structures. The mean number of metaphases was estimated. The background of breaks was subtracted from the breaks induced by 2 Gy. A B/M value of 0.46 is thus considered average, and dose reduction is recommended for B/M values above 0.55.

### 2.5. Statistical Analysis/Methods

The statistical analysis of the data was performed with SPSS Statistics 22 (IBM, Armonk, NY, USA). Statistical significance was tested by Mann–Whitney U and the T- and the Levene-test. The Graphs were plotted with Prism (9.5.1.) (GraphPad Software, San Diego, CA, USA).

## 3. Results

### 3.1. Prevalence of Cancer and Potential Underuse of Radiochemotherapy in SLE Patients

First, we studied whether radiotherapy is underused in patients with SLE with concomitant cancer (SLE-onco). Between 2000 and 2020, 1086 patients with SLE were treated at our University Hospital. The majority of patients were female (84.0%). The age at SLE diagnosis was 43.9 years in women and 50.9 years in men. Of these patients, we identified 64 patients with cancer (ICD10, C00-C97 without C44), most of whom were female (71.9%). The age at SLE diagnosis was higher in cancer patients with 52.7 years in females and 61.2 years in males. For 26 (40.6%) patients, the diagnosis of cancer was made on average 9.9 years prior to the SLE diagnosis and for 38 (59.4%) patients 2.8 years after the SLE diagnosis. Forty-three (67.4%) of SLE patients with cancer were treated with radio- or radiochemotherapy. In 15 (23.4%), RT was not indicated, in 5 (7.8%), therapy was feasible, and in 1 patient (1.6%), therapy was indicated but not performed. Thus, in our hospital, we found no underuse of RT in patients with SLE.

### 3.2. Approach to Radiosensitivity Testing

**Figure 1:**
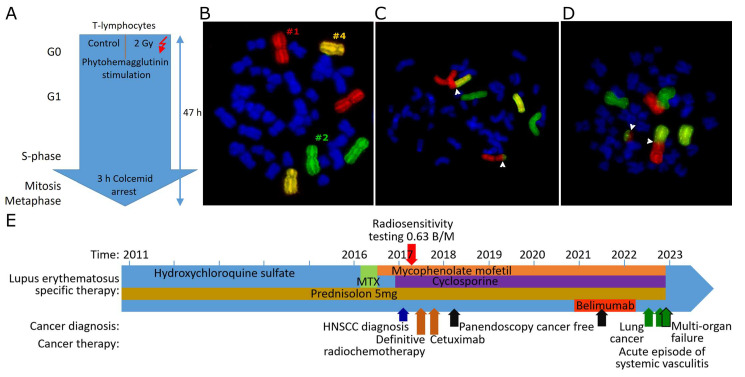
Fluorescence in situ hybridization and analysis of chromosomal aberrations scored as breaks per metaphase. (**A**) Scheme of treatment and stimulation of ex vivo blood for irradiation and generation of chromosomal aberrations. (**B**) Metaphase with red-stained chromosome #1, green chromosome #2, and yellow chromosome #4. (**C**) Chromosomes #1 and #2 involved in a translocation. (**D**) Chromosomes #1 and #2 involved in a dicentric aberration and an acentric. Translocations and dicentric aberrations are counted as two breaks per metaphase each. Break positions are marked by white arrowheads. (**E**) Treatment regimen of a radiosensitive patient with SLE. Timeline of SLE treatment (writing on the arrow) and treatment of an HNSCC and lung cancer (below the arrow).

The next step was to examine whether SLE patients are more sensitive to radiation. Therefore, individual radiation sensitivity was studied using three-color fluorescence in situ hybridization. Two ex vivo blood samples were used; one sample was not irradiated as a control and one was irradiated with a dose of 2 Gy ionizing radiation. The G0 phase lymphocytes in the blood were stimulated to divide and had 47 h to go through the cell cycle. Lymphocytes were stopped in metaphase with colcemid (Figure 1A), and chromosomes 1, 2, and 4 were painted with whole chromosome probes (Figure 1B). The chromosomal aberrations (Figure 1C,D) were scored for the breaks behind the aberrations. The breaks counted are expressed as breaks per metaphase (B/M) and indicate the number of DNA double-strand breaks underlying the chromosomal aberrations and are related to radiosensitivity.

### 3.3. A Case of a Radiosensitive Patient with HNSCC and SLE

To illustrate increased radiosensitivity in SLE, we present a case of a 57-year-old patient who was first diagnosed with SLE in 2011. He was highly positive for anti-SS-A and anti-cardiolipin antibodies. The main clinical manifestation of SLE is the effect on the skin. The patient was treated with hydroxychloroquine for six years, followed by two years of methotrexate. After an exacerbation of the disease during the methotrexate treatment and after an elevation of liver enzymes, the therapy was switched to mycophenolate mofetil and cyclosporine (Figure 1E). The patient had been a heavy smoker for many years (>20 pack years). In 2017, he was diagnosed with oropharyngeal squamous cell carcinoma (cT1 pN1 (1 out of 36 lymph nodes was positive) without extranodal extension M0, G3). The tumor board recommended radiochemotherapy due to the uncertainty of submucosal extension. The individual radiosensitivity of the patient was determined by 3C FiSH and was found to be elevated at 0.63 B/M. 0.46 B/M is considered average, and dose reduction is recommended for B/M above 0.55. The dose was reduced by 13% from 1.8 Gy to 1.6 Gy per fraction. The total dose was recommended to be reduced from 72 Gy to 64 Gy. The treatment had to be discontinued after 18 fractions and a total dose of 28.8 Gy due to a massive worsening of SLE in the form of a pronounced hemorrhagic oral mucositis and dermatitis. The anti-epidermal growth factor receptor monoclonal antibody cetuximab was administered every 7 days. No cancer cells were detected in two panendoscopic explorations 8 months and 4 years after definitive radiotherapy. More than five years later, the patient developed lung cancer and died of multiple organ failure, but there was no recurrence of oropharyngeal cancer.

### 3.4. Radiation Sensitivity Testing of SLE Patients and SLE-Oncology Patients

We were interested in the individual radiosensitivity of subjects with SLE. Individual radiosensitivity was studied in a cohort of 37 patients with systemic lupus erythematosus who did not have cancer (SLE-non-onco cohort) (mean age 49.6 years) and 33 patients with cancer and SLE (SLE-onco cohort) (56.6 years). The breaks per metaphase (B/M) of both cohorts were compared with 215 healthy individuals with an average age of 50.8 years and two cancer control groups with advanced rectal cancer (n = 226) with an average age of 62.8 years and with breast cancer with an average age of 57.3 years (n = 147). The SLE-non-onco cohort was 75.7% female and the SLE-onco cohort was 81.8% female. The diagnosis of SLE was made at a mean age of 36.7 years in the SLE-non-onco group and at a mean age of 48.0 years in the SLE-onco cohort. SLE-non-onco was diagnosed at 13.9 years and SLE-onco at 12.8 years prior to radiosensitivity testing. The clinical characteristics, symptoms, and therapy are shown in Table 1 and Table 2. The most common cancer types in the SLE-onco cohort included breast cancer (n = 15), lung cancer (n = 6), prostate cancer (n = 2), brain cancer (n = 2), anal cancer (n = 2), and others (n = 6) (Table 3).

### 3.5. Background Rates and Mutations After Radiation Are Increased in Oncologic Patients with SLE

**Figure 2:**
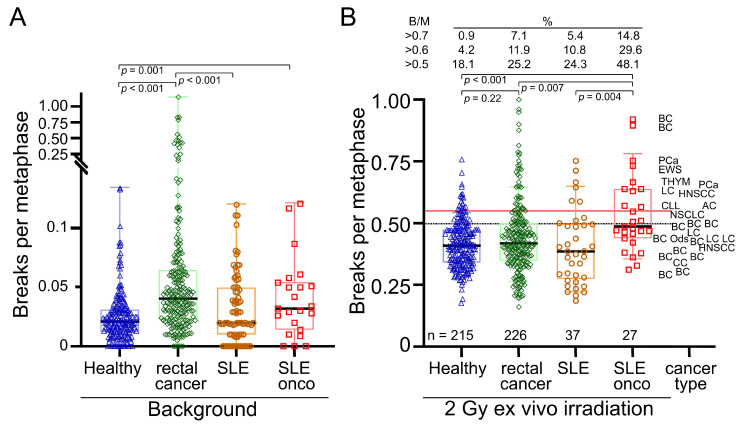
Chromosomal aberrations scored as breaks per metaphase (B/M). (**A**) Background values for breaks per metaphase in a cohort of 215 healthy individuals, 226 patients with rectal cancer, 37 patients with SLE, and 33 patients with SLE and cancer. (**B**) Breaks per metaphase after ex vivo irradiation with 2 Gy ionizing radiation and background correction and associated cancer types. Black horizontal lines indicate median values. Brackets indicate significant differences below *p* < 0.05. Percentage of individuals with values above 0.5, 0.6, and 0.7 breaks per metaphase in their cohort. BC = breast cancer, LC = Lung cancer, PCa = prostate cancer, HNSCC = head and neck squamous cell carcinoma, EWS = Ewing sarcoma, THYM = Thymoma, CLL = chronical lymphatic leukemia, Brain = cancer of the brain, CC = cervical cancer, AC = anal cancer.

We first compared the background rates of chromosomal aberrations in the healthy subjects, the rectal and breast cancer patient groups, and the two SLE patient groups to assess whether SLE patients have increased radiosensitivity. Background rates may be related to environmental exposures but also a reduced ability to adequately deal with DNA damage leading to the accumulation of mutations of the chromosomal aberration type. Background rates were clearly increased in the SLE-onco cohort (0.044 B/M ± 0.034) compared to healthy subjects (0.025 B/M ± 0.021, *p* = 0.001) (Figure 2A), while the rates in the SLE-non-onco group (0.032 B/M ± 0.029) had a radiosensitivity similar to the healthy control group. Radiosensitivity was also increased in the cohort of patients with rectal cancer (mean 0.078 B/M ± 0.142).

Next, we examined the residual aberrations after 2 Gy ex vivo irradiation and background reduction, scored as B/M. After 2 Gy irradiation there was no clear difference between the healthy (0.42 B/M ± 0.096) and SLE-non-onco cohort (0.40 B/M ± 0.150), while the SLE-onco (0.52 B/M ± 0.148) had an increased B/M (*p* < 0.007) (Figure 2B). The rectal cancer (0.44 B/M ± 0.146) and breast cancer (0.45 B/M ± 0.120) cohorts were not significantly different from the healthy control group. Regarding the outliers showing DNA breaks higher than 0.5, 0.6, or 0.7 B/M, the proportion of patients in the SLE-non-onco group was higher than that of the healthy individuals. SLE-onco had by far the highest proportion of outliers by 45.5% > 0.5 B/M, with nearly twice as many as the rectal cancer patients (25.2%) or the SLE-non-onco group (24.3%). Therefore, oncology patients with SLE have increased background rates and additionally an increased rate of DNA breaks after ex vivo irradiation as an indicator of increased radiosensitivity.

### 3.6. Male Sex but Not Age or the Type of Cancer Are Associated with Increased Radiosensitivity

**Figure 3:**
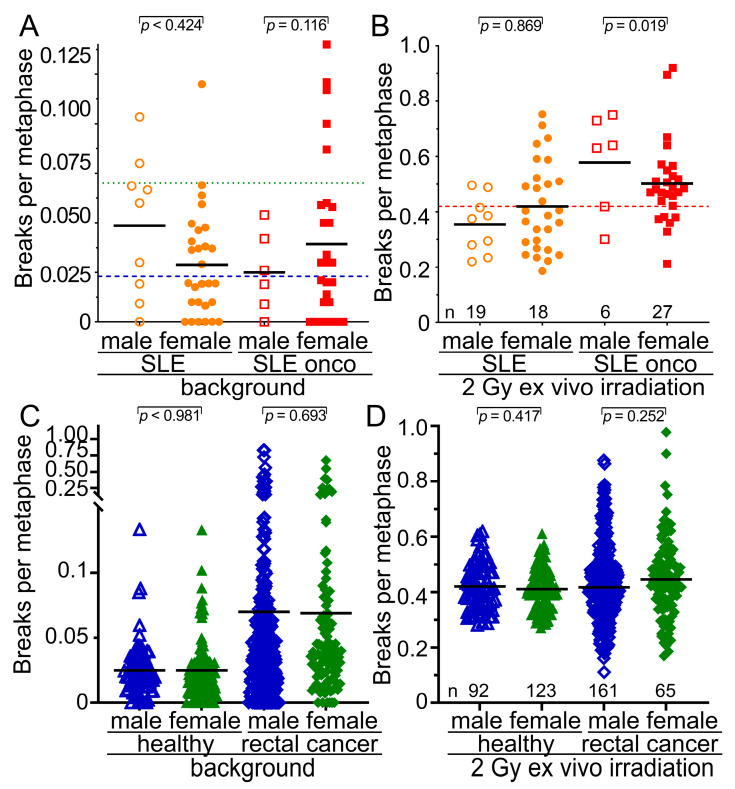
Relationship of radiation sensitivity to sex in the healthy, rectal cancer, and SLE cohorts. (**A**) Background values for breaks per metaphase in the cohort of 37 individuals with SLE and 28 individuals with SLE and cancer separated by sex. (**B**) Radiosensitivity after 2 Gy of ex vivo irradiation in the cohort of 37 individuals with SLE and 28 individuals with SLE and cancer separated by sex. (**C**) Background values for breaks per metaphase in the cohort of healthy subjects and rectal cancer patients divided by sex. (**D**) Radiosensitivity after 2 Gy of ex vivo irradiation in the cohort of healthy subjects and rectal cancer patients divided by sex. The blue long dashed horizontal line depicts the mean background breaks per metaphase of the healthy subjects and the green long dotted line the background breaks of the rectal cancer patients. The red long dashed horizontal line represents the mean number of breaks per metaphase for both healthy and rectal cancer subjects. The mean values are represented by the black short horizontal lines.

**Figure 4:**
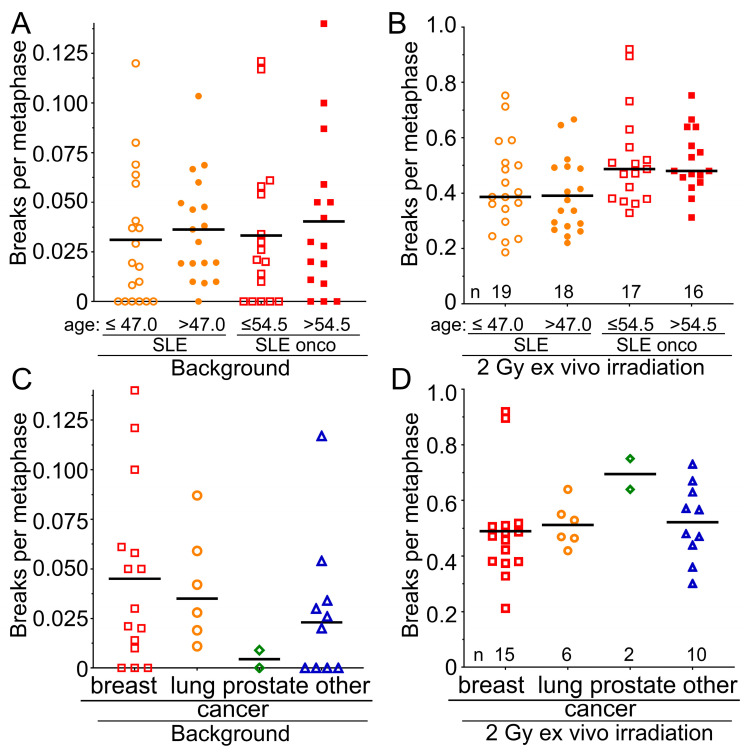
Dependency of radiation sensitivity on the age and the type of tumor in the SLE cohorts. (**A**) Background values for breaks per metaphase in the cohort of 37 individuals with SLE, separated by median age, younger than or equal to 47.0 years, and older than 47.0 years, and in the oncologic SLE cohort of 28 individuals, separated by median age, younger than or equal to 54.5 years. (**B**) Radiosensitivity after 2 Gy of ex vivo irradiation and according to the previous separations. (**C**) Background values for breaks per metaphase in the SLE-oncology cohort of 28 individuals, separated by breast, lung, prostate, and other cancers. (**D**) Radiosensitivity after 2 Gy ex vivo irradiation in the SLE-oncology cohort of 28 subjects, separated by breast, lung, prostate, and other cancers.

We were interested in determining further risk factors for increased radiosensitivity in the SLE cohort. We found that increased background values for breaks per metaphase occurred nearly twice as often in male patients in the SLE-non-onco and in the SLE-onco cohort (SLE-non-onco 0.0486 B/M; SLE-onco 0.050 B/M) compared to the female group (SLE-non-onco 0.0288; SLE-onco 0.0315 B/M) (Figure 3A). Oncologic SLE males have significantly increased radiosensitivity compared to females (*p* = 0.019) (Figure 3B). In the healthy controls and rectal cancer patients, we did not find any sex differences neither in background aberrations (Figure 3C) nor in the radiosensitivity (Figure 3D) between males and females (*p* > 0.252). In all cohorts, neither increased background chromosomal aberrations (Figure 4A) nor radiosensitivity (Figure 4B) were associated with age (*p* > 0.578). There was no statistical difference in background levels (Figure 4C) among the different examined types of cancer (*p* > 0.461). The most radiosensitive cancers were prostate cancers, yet these were only diagnosed in two individuals (*p* = 0.132) (Figure 4D). Hence, male sex is associated with increased radiosensitivity in SLE patients, while age and the type of cancer is not a risk factor for increased radiosensitivity.

### 3.7. Ro-52-Antibodies Are Associated with Increased Radiosensitivity

**Figure 5:**
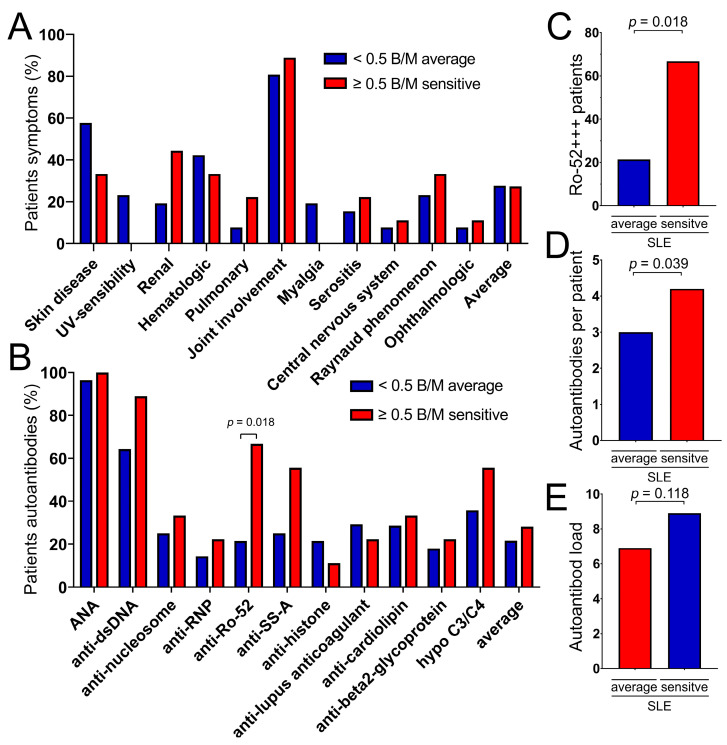
Antibodies and symptoms in the non-oncologic SLE cohort of 8 patients with increased radiosensitivity (red bars) were compared to the 29 patients with average radiosensitivity (blue bars). (**A**) Various symptoms of SLE in the average and in the sensitive group. (**B**) Various autoantibodies in the average (blue bar) and in the sensitive group (red bar). (**C**) Ro-52 strongly positive patients in the average and in the sensitive group. (**D**) Average number of autoantibodies per patient in the average and in the sensitive group. (**E**) Autoantibody load in the average und in the sensitive group.

We further investigated why some patients with SLE are more sensitive to ionizing radiation and others are not. Thus, we studied whether certain characteristics of the patients were related to the increased B/M levels. We compared the 9 SLE patients with B/M levels above 0.5 B/M with the 28 patients with lower B/M levels. Although clinical manifestations were not significantly associated with high B/M levels (Table 3, Figure 5A), Ro-52 autoantibodies were significantly elevated in the radiosensitive group (*p* = 0.018) (Figure 5B,C). Some autoantibodies such as anti-DNA-Ab (*p* = 0.179) and anti-SS-A-Ab (*p* = 0.102) tended to occur more frequently in the group with increased radiation sensitivity (Figure 5B); however, this did not reach statistical significance, possibly due to the small number of patients in each group. Overall, radiosensitive SLE patients showed significantly more autoantibodies compared to the non-radiosensitive group (*p* = 0.039) (Figure 5D). Similarly, the autoantibody load was higher in the sensitive group (Figure 5E). Therefore, Ro-52-antibodies are associated with increased radiosensitivity, while radiosensitivity is not influenced by the clinical manifestations.

### 3.8. Drugs in the Radiosensitivity Groups Are Evenly Distributed

As drugs could increase radiosensitivity, we studied the medications in the different cohorts. Most drugs were used infrequently and no clear differences were observed between the groups. The only exceptions were hydroxy- and chloroquine, which were frequently used and may additionally induce radiosensitivity. They were used slightly more frequently in the group with normal radiosensitivity (64.3%) than in the increased radiosensitivity group (55.6%) (Table 2).

### 3.9. Dose Adjustment According to the Radiosensitivity Testing Is Feasible In Vivo

We further investigated the clinical applicability of our findings. In total, 33 SLE-onco patients were evaluated for individual radiosensitivity. Overall, 15 patients had a B/M > 0.5 and dose reduction was recommended in 11 patients (Table 3). Although 0.5 B/M was already considered an increased radiation sensitivity, dose reduction was not recommended until 0.55 B/M. Nevertheless, the dose was reduced by one fraction in two patients in this radiosensitivity range (Supplementary Table S1).

**Figure 6:**
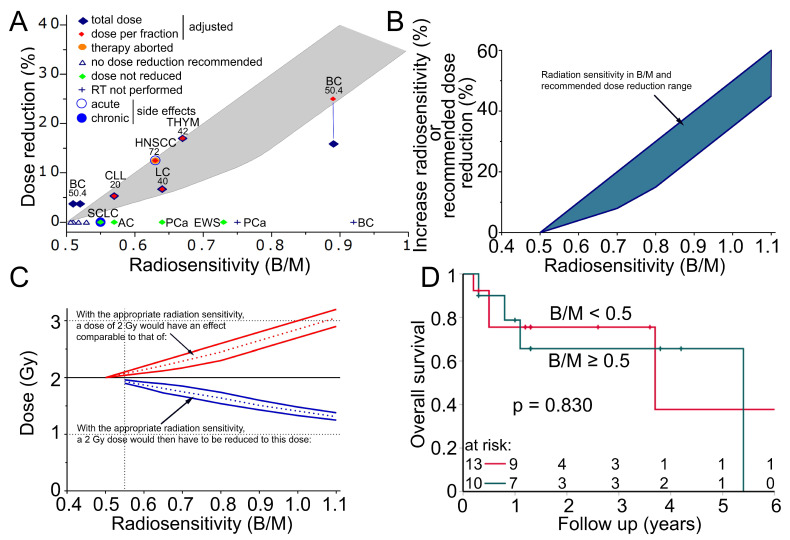
Radiation sensitivity in patients with systemic lupus erythematosus. (**A**) Fifteen patients with increased radiation sensitivity. Patients were recommended for dose reduction in the area of the gray envelope. Red diamond = dose per fraction was reduced at the given percentage, blue diamond = total dose was reduced at the given percentage, vertical blue line indicates that the dose per fraction and the total dose were reduced differently for the patient, orange dot = radiotherapy was aborted after 28 Gy due to exacerbation of SLE, green diamonds = dose was not reduced, blue cross = intended radiotherapy was not performed, blue circle = acute side effect occurred, blue dot = chronic side effect occurred. Cancer type and the intended dose (Gy) are indicated above each patient. BC = breast cancer, AC = anal carcinoma, LC = lung cancer, SCLC = small cell lung cancer, PCa = prostate cancer, HNSCC = head and neck squamous cell carcinoma, THYM = Thymoma, CLL = chronic lymphatic leukemia, EWS = Ewing’s sarcoma. (**B**) Empirically determined correlation between radiation sensitivity and increased radiation sensitivity in percent or recommended dose reduction with the estimated range of uncertainty (blue area). (**C**) The increased effect of a 2 Gy fraction is expected from the increased radiation sensitivity (red lines, dotted line mean value, solid line uncertainty range). The required reduction of a 2 Gy fraction at an increased radiation sensitivity to achieve an effect equivalent to the 2 Gy fraction (blue lines, dotted line mean value, solid line uncertainty range). (**D**) Kaplan–Meier plot of overall survival in 23 patients with systemic lupus erythematosus and concomitant oncologic disease. Overall, 10 patients with an increased radiosensitivity greater than 0.5 B/M were compared with 13 patients with average radiosensitivity.

At 0.55 B/M, a dose reduction in the range of 2–5% was recommended. A 35–50% dose reduction was recommended for patients with a B/M above 0.92 (Figure 6A). Treating physicians did not reduce the dose in four patients for whom a dose reduction was recommended. In five patients, the dose fractions were reduced within the recommended range. The total dose was reduced distinctly less than recommended in one of these patients with a high B/M of 0.90. One patient with prostate cancer (0.75 B/M) and one patient with breast cancer (0.92 BM) with relatively high radiosensitivity did not receive their intended radiotherapy and instead received an alternative treatment. To determine the increased radiation sensitivity, a calibration curve was developed using cells with known increased radiation sensitivity from patients with Ataxia Telangiectasia Mutated and Nijmegen Breakage Syndrome and experience with reduced doses in patients (Figure 6B). These estimates can then be used to determine how much higher a dose would be effective and by how much the dose for the radiation treatment should be reduced accordingly (Figure 6C). The overall survival of SLE-onco radiosensitive patients compared to non-radiosensitive patients was assessed using Kaplan–Meier analysis. Recruitment took place between December 2018 and October 2023. Follow-up was 2.3 years and 7 out of 23 patients died during this period. Increased radiosensitivity ≥ 0.5 B/M was used as the cut-off. Radiation-sensitive patients with a B/M ≥ 0.5 did not show a difference in overall survival compared to patients who did not have increased radiation sensitivity (*p* = 0.830) (Figure 6B). None of the SLE-onco patients experienced locoregional relapse within a follow-up of 2.1 years. Two patients, one with breast cancer and one with chronic lymphocytic leukemia (CLL), developed distant metastases. Of the sensitive patients (>0.55 B/M), the patient described in the case report experienced a serious acute adverse event, and a mildly sensitive patient who did not receive a dose reduction experienced ischemic colitis as a chronic adverse event.

## 4. Discussion

In this study, we present the analysis of radiosensitivity in two cohorts of SLE patients, one without cancer and one with cancer. When we compared the SLE cohort with a group of healthy controls, we found that a subgroup of SLE patients had an elevated background rate of breaks per metaphase and, additionally, an elevated rate of breaks per metaphase after ex vivo irradiation. In SLE patients who also suffer from cancer, the likelihood of having an increased sensitivity to radiation is even higher compared to healthy controls. SLE-onco patients had by far the highest number of exceptionally high radiosensitivity. When searching for risk factors for increased radiosensitivity, we found that the male sex and the presence of Ro-52-antibodies were associated with increased radiosensitivity in SLE-non-onco patients. An important aspect of this study in our SLE cohort is that dose adjustment was performed after radiosensitivity testing and was feasible without increasing the risk of relapse.

Increased sensitivity to radiation in patients with SLE has long been discussed in both cellular experiments and clinical observations. There are conflicting results in the cellular studies using different assays. In peripheral blood lymphocytes using the alkaline comet assay, 24 patients with juvenile SLE were more radiosensitive than controls [30]. In one study using the clonogenic assay, 14 SLE patients had increased radiosensitivity [12] while in another study only SLE patients in the active phase showed increased sensitivity [11]. After remission of SLE, the increased radiosensitivity disappeared.

Similar to our results, previous analyses showed that initial DNA double-strand breaks (DSBs) in lymphocytes of 52 SLE patients without cancer were not different from controls [14]. In a study on DNA DSB repair in lymphocytes of six SLE patients, each with active and quiescent disease, a reduced repair capacity was found [31]. This is consistent with our work in that the chromosomal aberrations are a consequence of unrepaired or incorrectly repaired DNA DSB. However, only a subgroup of SLE subjects were radiosensitive and we did not find a correlation with the anti-dsDNA ABs, although they were present in 65.4% of the SLE patients and 88.9% of the radiosensitive SLE patients. However, in our cohort, male sex and the presence of Ro-52-antibodies were associated with increased radiosensitivity in SLE patients. Most authors suggest that DNA repair is impaired in patients with SLE. One explanation for the increased radiosensitivity in some SLE patients may be that epigenetically regulated dysfunctions of DNA repair mechanisms occur [16]. Increased endogenous DNA damage, in part due to the induction of oxidative stress, may lead to increased accumulation of DNA [31]. As the repair capacity is only reduced if certain repair pathways are affected, only individual patients would have increased radiation sensitivity. This would depend on which repair pathways are affected. Changes in the nucleotide excision repair pathway would certainly hardly change the radiation sensitivity. Whereas, damage caused by ionizing radiation, such as DNA double-strand breaks, must necessarily be repaired by the non-homologous end joining. Impairment of the non-homologous end joining would certainly increase radiation sensitivity.

As increased radiation sensitivity results in a higher risk of adverse therapeutic effects, it is important to identify patients at risk. A number of case reports have described an increased risk of toxicity of radiotherapy in connective tissue disease, especially in SLE [7,10,13]. In a study of 209 patients with rheumatic diseases, no increased late side effects were found in patients with rheumatoid arthritis. Similarly, we found limited increased radiation sensitivity in RA with our three-color FISH test [32]. In a study with patients with connective tissue diseases, including 25 patients with SLE, radiotherapy was associated with an increased incidence of significant late side effects in 17% of the patients, and a cautious indication for radiotherapy was recommended [33]. No acute toxicities of radiotherapy were observed in a study of 86 patients with connective tissue disease, including 13 SLE patients, but increased late toxicities and particularly severe side effects were observed. SLE patients had the highest rates of side effects [34]. A retrospective study of 26 patients found a moderate risk for patients with SLE, which should not discourage therapy. A meta-analysis of 405 connective tissue disease patients found a significantly small increased risk of acute side effects and a more than twofold increased risk of late side effects in the 71 SLE patients [19]. On the other hand, 12 SLE patients with cervical cancer treated with modern intensity-modulated radiation therapy and brachytherapy tolerated radiotherapy well [35]. A further study of 197 connective tissue disease patients, including 34 patients with SLE, treated with modern radiotherapy showed that no patients with systemic lupus erythematosus developed severe toxic effects after receiving moderate hypofractionation (>2 Gy to <5 Gy per fraction) or ultrahypofractionation (>5 Gy per fraction) radiotherapy [36]. One paper notes that of 26 patients for whom RT was recommended, only 4 patients received radiotherapy. None of the four developed toxicity. They concluded that patients are inadequately deprived of radiotherapy.

In our prospective study of sensitive patients (>0.55 B/M), one patient experienced an acute adverse event and one patient who was mildly sensitive but did not receive a dose reduction experienced a chronic adverse event. We found no underuse of radiotherapy in our hospital in the retrospective part of the study from 2000 to 2020. However, this is a single-institution study and may be handled differently in other hospitals [37].

In summary, based on the data from previous clinical trials and the results of our study, there is an increased risk of late side effects in a subgroup of patients with SLE. Advanced SLE, high radiation doses, and irradiation of organs at risk increase the risk of side effects, however, with the use of modern radiotherapy and careful selection of patients RT in patients with SLE, should be possible [26].

SLE is a complex disease for which the pathogenesis is not clearly known [38]. However, it is clearly linked to the presence of autoantibodies [1]. Autoantibodies appear to be associated with cancer risk in SLE [39,40,41,42]. Cancer (26.7%) was among the most common causes of death in SLE, along with infections (31.7%) and cardiovascular disease (21.8%) [43]. There is a decreased risk for certain tumor types and an increased risk for others [3,44]. In breast cancer, the cancer ratio is clearly decreased in SLE patients and this is associated with the anti-DNA autoantibodies [40,41,42,44]. This is similar for endometrial, and possibly ovarian and prostate, cancers [35,39]. An increased risk is assumed for hematologic, lung, thyroid, liver, bladder, pancreatic, kidney, nasopharyngeal, and HPV-associated (vaginal, vulvar, cervical, anal) malignancies [3]. A breast cancer study identified dsDNA, lupus anticoagulans, and anticardiolipin autoantibodies in 2431 SLE patients associated with a reduction in cancer incidence [44]. First, one might tend to associate a lower cancer risk with reduced radiosensitivity. However, it is probably the higher autoantigenicity of cancer cells in SLE and the resulting inflammation, rather than the lower cancer induction, that is responsible for the lower cancer risk.

Increased radiation sensitivity does not necessarily lead to an unfavorable therapeutic outcome. A distinction must be made between increased and very high radiation sensitivity. Increased radiation sensitivity in our system ranges from 0.5 B/M to approximately 0.75 B/M. A patient with increased radiation sensitivity has a limited increased risk of experiencing an adverse event over time. Additionally, if the overall risk of therapeutic consequences is already low in the case of radiation therapy, such as breast irradiation, this risk remains low even in the case of increased radiation sensitivity. In the case of more intense therapy, such as head and neck cancer, where there is already a higher risk of late side effects from the therapy, increased radiation sensitivity will lead to even more radiation effects. In this area of moderately increased radiosensitivity, the consequences of radiation must be regarded as stochastic events and therefore there is only a probability that the consequences of therapy will occur. In our measurements, on the other hand, the highly increased radiation sensitivity starts at about 0.8 B/M–3 B/M. This includes extreme radiosensitivities in homozygous pathogenic variants of Nijmegen breakage syndrome or ataxia-telangiectasia. In these cases, there is such a strong increase in radiation sensitivity that severe unwanted radiation effects are inevitable with any normally performed radiation therapy. However, there are also patients who have not been previously assigned to any syndrome but who are extremely sensitive to radiation in our system > 0.8 B/M. We usually see these in retrospective studies when patients have suffered a serious consequence of therapy. These events must be regarded as deterministic events where it is certain that the event will occur.

Dose adjustment should be considered in patients with increased radiation sensitivity. Patients with severely increased radiosensitivity should have their dose adjusted. In our study, the fractional dose was mostly reduced. This seems to be much better than just reducing the total dose. We argue that a reduced fraction is then just as effective for the radiosensitive patient as a normal fraction for a patient of average sensitivity. If only the total dose is reduced, each individual dose is more effective due to the non-linear dose-response relationship, and the effect of the dose reduction is difficult to estimate. Dose reduction has been successfully used in genetic diseases, such as in a patient with Phelan-McDermid syndrome [45]. A small number of SLE patients were doses adjusted in this study, which appears to have been successful. Although we consider people with 0.5 B/M to have increased radiosensitivity, we recommend dose reduction only from 0.55 B/M. In this low range of sensitivity, the risk of adverse effects is very low and only a very small dose adjustment would be required. However, four recommended dose reductions were not implemented because the treating physicians were concerned about under-dosing the cancer and finally did not trust the concept of radiation sensitivity. It is clear that the higher the chromosomal sensitivity, the less certain it is as to how much the dose should be reduced. It is therefore understandable that two patients who were eligible for alternative therapy chose this option. In one patient with a high sensitivity of 0.9 B/M, the fractional dose was reduced more than the total dose. There is also a possibility if one is concerned about tumor control, a greater reduction in the fractional dose will make the therapy more tolerable and still have a greater effect on tumor control.

An important prerequisite for dose adjustment based on radiation sensitivity in normal tissue is that there must also be increased radiation sensitivity in the tumor. This is evident in genetic diseases, as the tumor will inevitably contain the corresponding genetic variants [46]. In the case of SLE, where it is not clear how the increased radiosensitivity arises, this situation is more complex. The case example in this study, where a head and neck tumor can be considered cured with a relatively low dose, is probably an indication of it. The successful dose adjustments in our study, despite the limitations, suggest that the increased radiation sensitivity of normal tissues in patients with SLE also influences the radiation sensitivity of the tumor.

A limitation of this study was the small number of SLE patients with dose reductions and the relatively short follow-up period. Another limitation is that patients on high doses of cortisone (prednisolone > 20 mg/d) cannot be studied with our assay because the lymphocytes can no longer be stimulated to enter the cell cycle and therefore no metaphases are available. It is possible that this group with high disease activity may also have an increased sensitivity to radiation. Unfortunately, this could not be studied with our assay. An important advantage of the method we use is that we measure DNA damage, i.e., mutations in the form of chromosomal aberrations. Another advantage is that the influence of the patient’s toxic environment is minimal because we subtract the background of existing aberrations from the aberrations produced by 2 Gy.

## 5. Conclusions

Among oncologic SLE patients, there is a high proportion of individuals at risk for significantly increased radiosensitivity. Radiation sensitivity testing should be performed prior to radiation therapy or patients should be monitored more closely during therapy to adjust the radiation dose and reduce the risk of side effects.

## Figures and Tables

**Table 1:** Clinical characteristics and SLE manifestations of the SLE-non-onco group and the SLE-onco group. Missing data are in square brackets.

	Involved (%) [No Data]	
Symptoms	SLE-Non-Onco Group	SLE-Onco Group
	*n* = 37	*n* = 33
Mean age at radiosensitivity testing, years (range)	49.6 (22–78)	56.6 (47–80)
Sex (female/male)	28/9 (75.7/24.3)	27/6 (81.8/18.2)
Mean age at diagnosis of SLE (range)	36.7 (12–77)	38.0 (33–66)
Time between SLE diagnosis/cancer diagnosis (years)	---	13.8 (2–47)
Skin	18 (51.4%)	15 (83.3%) [18]
UV-sensibility	6 (17.1%)	0 (0%) [15]
Kidneys	9 (25.7%)	3 (16.7%) [15]
Hematopoietic System	14 (40%)	3 (15.8%) [14]
Lungs	4 (11.4%)	3 (16.7%) [15]
Arthritis/Arthralgia	29 (82.9%)	9 (50%) [15]
Myositis/Myalgia	5 (14.3%)	0 (0%) [15]
Serositis	6 (17.1%)	5 (27.8%) [15]
Central nervous system	3 (8.6%)	0 (0%) [15]
Raynaud phenomenon	9 (25.7%)	3 (16.7%) [15]
Eye	3 (8.6%)	5 (27.8%) [15]

**Table 2:** Medication in SLE-non-onco patients and in the subgroups of SLE-non-onco patients with normal radiosensitivity (B/M < 0.5) and increased radiosensitivity (B/M ≥ 0.5) at the time of testing. Percentage of patients in the different cohorts taking the listed drugs.

	SLE-Non-Onco Group Patients Taking Drugs (%)		
		Radiosensitivity	
Drug	All	Normal	Increased
	*n* = 37	*n* = 28	*n* = 9
Hydroxychloroquine	17 (45.9)	12 (42.9)	5 (55.6)
Chloroquine	7 (18.9)	7 (25.0)	0 (0)
Methotrexat	4 (10.8)	3 (10.7)	1 (11.1)
Azathioprine	5 (13.5)	3 (10.7)	2 (22.2)
Belimumab	6 (16.2)	4 (14.3)	2 (22.2)
Mycophenolat-Mofetil	3 (8.1)	1 (3.6)	2 (22.2)
Rituximab	1 (2.7)	1 (3.6)	0 (0)
Tacrolimus	1 (2.7)	1 (3.6)	0 (0)
Average number of drugs per patient	1.2	1.14	1.33

**Table 3:** Radiosensitivity, therapy, and outcome of patients in the SLE-onco cohort.

	Variable	SLE-Onco Patients (*n* = 33)
	Cancers (%)	BC: 15 (45.5) LC: 6 (18.2) Pca: 2 (6.1) Brain: 2 (6.1) Anal: 2 (6.1) EWS: 1 (3) THYM: 1 (3) HNSCC: 1 (3) CC: 1 (3) SC: 1 (3) CLL: 1 (3)
Radiosensitivity	Average B/M	0.516 ± 0.153
	Increased radiosensitivity	B/M < 0.5: 18 (54.5%); B/M 0.5–0.55: 4 (12.1%); B/M > 0.55: 11 (33.3%)
Radiotherapy performed:	Yes/no	30/3
Dose reduction	Recommended/done	11/not reduced: 4; reduced: 5; RT not performed: 2
	average dose reduction:	11%
Fractionation dose	Planned/performed	2.78 ± 1.71/2.67 ± 1.56
Total dose	Planned/performed	45.9 ± 13.89/43.4 ± 12.45
Follow up	Median/average (years)	1.3/2.1
Therapeutic outcome	Recurrence/Metastasis	0 (0%)/2 (6.7%)
acute side effects	Skin/others/beyond the normal	11 (42.1%)/8 (36.3%)/4 (19.0%)
	RT reactive intensification of the SLE	2 (9.5%)
chronic side effects	Skin/others/beyond the normal	4 (18.2%)/1 (4.8%)/3 (13.0%)

BC = breast cancer, LC = Lung cancer, PCa = prostate cancer, HNSCC = head and neck squamous cell carcinoma, EWS = Ewing’s sarcoma, THYM = Thymoma, CLL = chronic lymphatic leukemia, Brain = cancer of the brain, CC = cervical cancer, AC = anal cancer.

## Data Availability

The datasets generated and/or analyzed in the present study are not publicly accessible but can be provided by the corresponding author upon reasonable request.

## Supplementary Materials

The following supporting information can be downloaded at https://www.mdpi.com/article/10.3390/cells14080569/s1: Table S1: Characterization of the 33 SLE-onco cohort in terms of cancers, age, radiosensitivity, planned and performed fraction size and total dose, side effects, and immunosuppressive drugs.
