# Supplementary material for: Increased Sensitivity to Ionizing Radiation in a Relevant Subset of Patients with Cancer and Systemic Lupus Erythematosus
Source: Cells. 2025 Apr 9;14(8):569. doi: 10.3390/cells14080569 (PMC12025991; doi:10.3390/cells14080569)
Supplement: Supplementary file 1 [file cells-14-00569-s001.zip › cells-3549030-supplementary.pdf]

Table S1 Characterization of the 33 SLEonco cohort in terms of cancers, age, radiosensitivity, planned and performed fraction size and total dose, side effects, and immunosuppressive drugs.

			Fraction dose (Gy)		Total dose (Gy)			Side effects			reactivation of SLE			drugs	
							simultaneous	acute	chronic						
Cancer	age (years)	radio-sensitivity (B/M)	planned (boost)	performed (boost)	planned (boost)	performed (boost)	chemo therapy	during therapy	Skin	others	acute	chronic	distant metastasis	Steroids	Immuno suppressive treatment
BC	63	0.21	2.67	2.67	40.0	40.05	no	no	no	no	no	no	no	no	-
BC	46	0.33	1.8	1.8	50.4	50.4	no	no	no	no	no	no	no	no	-
BC	44	0.37	1.8	1.8	50.4	50.4	no	no	no	no	no	no	no	no	-
BC	66	0.38	1.8 (2)	1.8 (2)	50.4 (66)	50.4 (66)	no	no	no	no	no	no	no	prednisolone 40 mg	HCQ
BC	47	0.38	1.8	1.8	50.4 (71)	50.4 (66)	no	no	no	no	yes	no	primary metastasis	no	HCQ, MMF
BC	51	0.42	1.8	1.8	50.4	50.4	no	no	no	no	no	no	no	no	-
BC	60	0.46	2	2	50	50	no	breast edema 1	2	fatigue 1-2	no	no	no	no	HCQ
BC	50	0.47	1.8 (2.1)	1.8 (2.1)	50.4 (58.8)	50.4 (58.8)	no	1	1	no	no	no	no	no	-
BC	58	0.48	1.8	1.8	50.4	50.4	no	no	no	no	no	no	no	no	-
BC	54	0.49	1.8 (2)	1.8 (2)	50.4 (66)	50.4 (66)	no	no	no	no	no	no	no	no	no
BC	41	0.51	1.8	1.8	50.4	50.4	no	no	no	no	no	no	no	no	-
BC	53	0.51	1.8	1.8	50.4	**48.6**	no	1	1	no	no	no	no	prednisolone 5 mg	HCQ
BC	45	0.52	1.8	1.8	50.4	**48.6**	no	no	no	no	no	no	no	no	-
BC	39	0.89	2	**1.6**	50	**47.2**	no	no	no	no	no	probably	no	no	HCQ
BC	49	0.92		no radiotherapy. Mastectomy		-	-	-	-	-	-	-	no	prednisolone	Ciclosporin A
LC	75	0.42	3	3	60	60	no	no	no	no	no	no	no	no	-
LC	62	0.46	5	5	50	50	no	1	no	no	no	no	no	no	-
LC	64	0.47	3	3	60	60	yes	no	1	no	no	no	no	prednisolone	HCQ
LC	71	0.53	3	3	60	60	yes	no	no	no	no	no	no	no	-
LC	58	0.55	3	3	30	30. 11th rib mediastinum and whole brain	no	1	no	5	no	no	no	no	HCQ

LC	68	0.64	8	**7.5**	40	**37.5**	no	no	no	no	no	no	no	triamcinolone	-
PCa	80	0.64	1.5 (1.6, 1.8)	1.5 (1.6, 1.8)	46.5 (58.8, 66)	46.5 (58.8, 66)	no	no	no	no	no	no	no	no	no
PCa	64	0.75	no radiotherapy. surgery			-	-	-	-	-	-	-	no	no	no
Brain	50	0.47	1.8	1.8	59.4	59.4	CCNU	no	no	no	no	no	no	no	-
Brain	66	0.48	20	20	20	20	no	no	no	no	no	no	no	prednisolone	HCQ
Anal	42	0.30	1.8	1.8	54	54	yes	no	no	no	no	no	no	no	-
Anal	53	0.57	1.8	1.8	54	54	yes	1	no	no	no	no	no	no	-
EWS	53	0.73	1.6 (1.8)	1.6 (1.8)	40 (54)	40 (54)	no	no	no	no	no	no	no	no	no
THYM	55	0.67	6	**5.1**	42	**35.7**	no	no	no	no	no	no	no	prednisolone	Rituximab
HNSCC	54	0.63	1.8	**1.6**	72	**28.8***	yes	2	no	no	yes	yes	no	no	HCQ, MMF
CC	47	0.36	1.8	1.8		44<br>40Gy PDR	Cis. switched to Carbo.	Ischemic colitis 5	no	no	no	no	no	no	-
SC	70	0.44	2	2	50	50	no	1	no	no	no	no	no	no	HCQ
CLL	63	0.57	2	1.9	20	**19**	no	no	no	no	no	no	yes	no	HCQ

* = canceled due to exacerbation of SLE

Abbreviations: Toxicity score according to CTCAE version 5.0. BC = breast cancer, LC = Lung cancer, PCa = prostate cancer, HNSCC = head and neck squamous cell carcinoma, EWS = Ewing sarcoma, THYM = Thymoma, CLL = chronical lymphatic leukemia, Brain = cancer of the brain, CC = cervical cancer, AC = anal cancer, HCQ = Hydroxychloroquine, MMF = mycophenolate mofetil;
